# Effect of pharmacological treatment of attention-deficit/hyperactivity disorder on later psychiatric comorbidity: a population-based prospective long-term study

**DOI:** 10.1136/bmjment-2024-301003

**Published:** 2024-09-19

**Authors:** Ingvild Lyhmann, Tarjei Widding-Havneraas, Ingvar Bjelland, Simen Markussen, Felix Elwert, Ashmita Chaulagain, Arnstein Mykletun, Anne Halmøy

**Affiliations:** 1 Centre for Research and Education in Forensic Psychiatry, Haukeland University Hospital, Bergen, Norway; 2 Department of Clinical Medicine, University of Bergen, Bergen, Norway; 3 Division of Psychiatry, Haukeland University Hospital, Bergen, Norway; 4 Ragnar Frisch Centre for Economic Research, Oslo, Norway; 5 Department of Sociology, University of Wisconsin-Madison, Madison, Wisconsin, USA; 6 Department of Biostatistics and Medical Informatics, University of Wisconsin-Madison, Madison, Wisconsin, USA; 7 Department of Population Health Sciences, University of Wisconsin-Madison, Madison, Wisconsin, USA; 8 Centre for Work and Mental Health, Nordlands Hospital, Bodø, Norway; 9 Division of Health Services, Norwegian Institute of Public Health, Oslo, Norway; 10 Department of Community Medicine, University of Tromsø, Tromsø, Norway

**Keywords:** Child & adolescent psychiatry, Impulse control disorders, Substance misuse, Depression & mood disorders, Anxiety disorders

## Abstract

**ABSTRACT:**

**Background:**

Psychiatric comorbidity is frequent among persons with attention-deficit/hyperactivity disorder (ADHD). Whether pharmacological treatment of ADHD influences the incidence of psychiatric comorbidity is uncertain.

**Objective:**

To investigate associations and causal relations between pharmacological treatment of ADHD and incidence of subsequent comorbid psychiatric diagnoses.

**Methods:**

We employed registry data covering all individuals aged 5–18 years in Norway who were diagnosed with ADHD during 2009–2011 (n=8051), followed until 2020. We used linear probability models (LPM) and instrumental variable (IV) analyses to examine associations and causal effects, respectively, between pharmacological treatment and subsequent comorbidity.

**Findings:**

From time of ADHD diagnosis to 9 years of follow-up, 63% of patients were registered with comorbid psychiatric disorders. For males, LPM showed associations between ADHD medication and several incident comorbidities, but strength and direction of associations and consistency over time varied. For females, no associations were statistically significant. IV analyses for selected categories isolating effects among patients ‘on the margin of treatment’ showed a protective effect for a category of stress-related disorders in females and for tic disorders in males for the first 2 and 3 years of pharmacological treatment, respectively.

**Conclusions:**

Overall, LPM and IV analyses did not provide consistent or credible support for long-term effects of pharmacological treatment on later psychiatric comorbidity. However, IV results suggest that for patients on the margin of treatment, pharmacological treatment may initially reduce the incidence of certain categories of comorbid disorders.

**Clinical implications:**

Clinicians working with persons with ADHD should monitor the effects of ADHD medication on later psychiatric comorbidity.

**Trial registration number:**

ISRCTN11891971.

**WHAT IS ALREADY KNOWN ON THIS TOPIC**

- Psychiatric comorbidity is prevalent among persons diagnosed with attention-deficit/hyperactivity disorder (ADHD). Knowledge about effects of pharmacological treatment of ADHD on subsequent psychiatric comorbidity is called for.

**WHAT THIS STUDY ADDS**

- We provide results based on a quasi-experimental design and detailed population-based data with long follow-up time. Medication for ADHD seems to protect against some types of comorbidities in the first years after initiation, although no convincing effects were found in the longer term, neither positive nor negative.

**HOW THIS STUDY MIGHT AFFECT RESEARCH, PRACTICE OR POLICY**

- Effects of ADHD medication on later psychiatric comorbidity should be considered with care in the treatment decision and follow-up.

## Introduction

 Attention-deficit/hyperactivity disorder (ADHD) is estimated to affect about 6% of children and adolescents^1^ and 3% of adults.^2^ Persons with ADHD commonly suffer from additional mental disorders, with prevalence varying from 40% to almost 90% in children and adolescents depending on the population studied.^3^ Such comorbid conditions may share risk factors with ADHD, or may be a consequence of the ADHD symptomatology.^4^ Either way, additional comorbid disorders will increase the total symptom load and further reduce the individual’s level of functioning.^5^

While the effect of pharmacological treatment on ADHD core symptoms of inattention, impulsivity and hyperactivity is well established,^6^ the extent to which this intervention alleviates real-life outcomes is not settled,^7^ and there is still some dispute about the ‘benefit to harm balance’ in the longer term.^8^ Regarding psychiatric comorbidity, medication may be potentially unfavourable for some mental symptoms, like tics and anxiousness,^9 10^ although a more recent meta-analysis and naturalistic prospective study challenge these findings.^11 12^

Despite comorbidity being the rule rather than the exception in the clinical ADHD population, comorbid disorders are most often an exclusion criterion in clinical trials studying the effects of ADHD medication.^13 14^ Furthermore, practically as well as ethically, randomised controlled trials are unfeasible to study the effects of long-term pharmacological treatment of ADHD in children and adolescents. Non-randomised naturalistic follow-up studies, on the other hand, will typically be limited by confounding bias. An alternative approach is to combine quasi-experimental methods with administrative register data that can span many years with practically no attrition. In Norway, a universal healthcare system and national guidelines for clinical practice are put in place to facilitate equal access to health services, irrespective of the citizen’s geographical residence or economic resources. Still, considerable geographical variation in ADHD diagnosis and treatment practices have been observed independent of variation in symptom load.^15^

While unwanted from a public health service perspective, such variation can be used for a quasi-experimental instrumental variable (IV) design.^16^ ^17^ We use variation in healthcare providers’ estimated preference for pharmacological treatment as a source of quasi-randomisation to treatment to estimate causal effects of ADHD medication on subsequent incidence of comorbid diagnoses.

### Objective

The objective of this study was to investigate the associations between pharmacological treatment of ADHD and the subsequent incidence of comorbid psychiatric diagnoses, and to establish whether possible associations were causal.

## Methods

### Data sources

All data were obtained in de-identified form from nationwide, individually linked, complete population registers in Norway.

### Sample

Our ADHD patient sample was defined as all persons in the Norwegian population who were aged between 5 and 18 and received an initial primary diagnosis of ADHD—corresponding to International Classification of Diseases-Tenth Revision (ICD-10) codes F90.0 (81.3%), F90.1 (11.3%), F90.8 (6.2%) and F90.9 (1.1%)—from the Norwegian Child and Adolescent Mental Health Services (CAMHS) during the years 2009–2011 (n=8051).

### Outcome

We defined psychiatric comorbidity as any mental or behavioural disorder diagnosed in addition to ADHD. We analysed the occurrence of incident psychiatric comorbidities, defined as disorders first diagnosed subsequent to the ADHD diagnosis. We had access to data on all ICD-10 Chapter V (‘Mental and behavioural disorders’, F00–F99) diagnoses registered in the CAMHS from 2009 to 2020. For feasibility reasons, and because of low prevalence of some of the disorders in children and adolescents, we grouped the relevant diagnoses into what we considered clinically meaningful categories of disorders: substance use disorder (SUD), psychotic, bipolar/other affective, depressive, anxiety, ‘reactive’ (including stress-related and somatoform disorders), sleep, eating, personality, pervasive developmental, specific developmental, conduct disorder (CD), tic, and various childhood disorders, and intellectual disability (ID). (See online supplemental table S1 for a detailed overview.)

Comorbidity was measured by counting the number of patients registered with one or more diagnoses within each disorder category separately. Possible multiple diagnoses within one category were thus not taken into consideration, while individuals registered with multiple comorbid diagnoses from different categories were counted multiple times.

For the multivariable analyses, we also created a summary diagnostic category, ‘any’, comprising all psychiatric comorbidities that are plausibly influenced by ADHD medication (hence excluding all developmental disorders and ID, based on authors’ clinical judgement) and are considered properly recorded in the Norwegian registers (hence excluding sleep disorders, which are clinically common^918^,^20^ but infrequently registered).

### Treatment

Pharmacological treatment was defined as filled prescriptions for any of the following ADHD medications (shares of total prescriptions in parentheses): methylphenidate (87.5%), atomoxetine (11.54%), dexamphetamine (0.8%), lisdexamphetamine (0.06%), amphetamine (0.04%) and guanfacine (none). (Clonidine is not approved for ADHD in Norway and only rarely used off-label.) Treatment was measured by the cumulative amount of prescriptions filled across the duration of follow-up (see details in the Descriptive and statistical analyses section), and scaled in defined daily doses (DDD) of prescriptions so that a one-unit increase represents ‘full-time’ pharmacological treatment for the period (eg, 2×365=730 DDDs of prescriptions filled across 2 years).

### Covariates

All analyses were adjusted for a wide range of covariates at the individual, family and catchment area level, such as sex, age at diagnosis, country of birth, prevalent comorbid psychiatric diagnoses at time of ADHD diagnosis, parental education and income, and area-level socioeconomic conditions. Complete overview is available in online supplemental table S2.

### Descriptive and statistical analyses

In the descriptive analysis, we investigated the extent of comorbidity at time of ADHD diagnosis and incidence over the following years. Next, we looked at relations between pharmacological treatment and incident comorbidity, that is, only comorbidity registered after the ADHD diagnosis. We estimated two classes of models. First, we ran conventional linear probability models (LPM) for the association between pharmacological ADHD treatment and incident comorbid psychiatric diagnoses over up to 9 years of follow-up. Follow-up was operationalised in terms of cumulative follow-up years after ADHD diagnosis, that is, time from ADHD diagnosis until 1 year later, time from ADHD diagnosis until 2 years later, etc. Models were stratified by sex and controlled for all baseline covariates, but do not control for possible unobserved confounding, for example, symptom severity, other medication use or healthcare utilisation (since these data were either inaccessible or of limited validity).

Second, we conducted two-stage least squares (2SLS) IV analyses to correct for possible unobserved confounding in the LPMs. Stratification and adjustment for potential confounding was similar for IV and LPM. IV analysis is a quasi-experimental technique that seeks to exploit the as-if random variation in treatment assignment that is induced by a so-called IV. We used the observed geographical variation in ‘provider preference’ (PP) for prescribing ADHD medication as IV, measured as the average number of DDDs for ADHD medication filled by patients with ADHD at each clinic during the defined follow-up period of each analysis (ie, over 1 year in analyses of medication effects in the first year after ADHD diagnosis, over 2 years in analyses of medication effects in the first 2 years, etc). Measurement of PP excluded the index patient (leave-one-out mean) to avoid reverse causality. Prior research suggests that PP for ADHD medication in Norway is plausibly as-if random in the sense of being unrelated to observed ADHD symptom load across geographical areas.^21^

Under standard IV assumptions,^22^ 2SLS estimates the local average treatment effect (LATE), that is, the average causal effect of medication for patients who take versus do not take medication due to their healthcare provider’s treatment preference. These patients are sometimes called the patients ‘on the margin’ of treatment, and do not include patients for whom there is no variation in clinicians’ medication decisions (ie, presumably those most lightly or heavily affected by ADHD symptoms). Self-selection to clinics with different PP is unlikely, as allocation is based on place of residence and switching clinics is highly unusual.

For an IV to be valid, several conditions must be met^23^: (1) *relevance*: the IV must predict treatment; (2) *exclusion:* the IV must not affect the outcome except through treatment; (3) *independence*: the IV shares no unobserved common causes with the outcome (ie, is as-if randomised to the patients conditional on covariates); (4) *monotonicity*: the IV may only affect treatment in one direction. While *relevance* can be empirically tested, the remaining assumptions must be defended based on theoretical reasoning and prior knowledge.^1722^,^24^

SEs in all models were clustered at the clinic level, and we report 95% CIs. We follow convention in comparing estimates of LPM and 2SLS^22^ to gauge the problem of unobserved confounding in the LPM models. To ensure that our results are robust to functional form assumptions, the main analyses were repeated using Probit and Probit-IV models, respectively. Data preparation, analysis and visualisation was performed in Stata V.17.

## Findings

### Incidence of comorbidities after ADHD diagnosis

**Figure 1:**
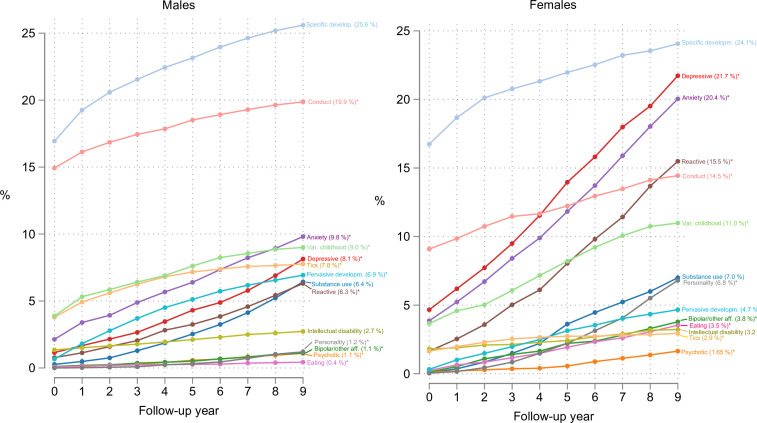
Cumulative incidence of comorbidity by years of follow-up. Year 0 signifies time of attention-deficit/hyperactivity disorder (ADHD) diagnosis; registered comorbid disorders at this baseline may include incident or prevalent cases.

Table 1 presents the baseline characteristics of our sample. Figure 1 shows the cumulative incidence of comorbid psychiatric disorders by category in the ADHD patient sample, by sex and per year from time of diagnosis to follow-up at 9 years (available in table format in online supplemental table S3). At the time of initial ADHD diagnosis, 37.4% of the ADHD patient group were registered with one or more additional psychiatric diagnoses. After 1 year, this number had risen to 43.0%; by 4 years, 51.8%; and by 9 years of follow-up, 62.7%.

**Table 1:** Baseline characteristics for patients diagnosed with ADHD in the CAMHS at ages 5–18 during 2009–2011

	Patients with ADHD (n=8051)
**Patient characteristics**	
Male, n (%)	5566 (69.1)
Age at diagnosis, mean±SD*	11.7±3.4
Males, mean±SD	11.3±3.3
Females, mean±SD	12.6±3.5
**Family characteristics**	
Parents with primary school education only, n (%)	
Mother	2640 (32.8)
Father	2561 (31.8)
Parents married, n (%)	
Mother	3785 (47.0)
Father	3767 (46.8)
Parents’ labour income (US$)†, mean±SD	
Mother	28 374±24 879
Father	54 900±40 410
**Catchment area characteristics**	
Population size, mean±SD	32 913±26 765
Parents with primary school education only, %±SD	7.9±4.6
Mothers married, %±SD	60.4±6.3
Parents’ labour income (US$), mean±SD	48 019±7192

* Plus-minus values are mean±standard deviations (SD).

† US$/NOK (Norwegian krone) exchange rate average for 2010 (US$1/NOK6.0453).

ADHD, attention-deficit/hyperactivity disorder; CAMHS, Child and Adolescent Mental Health Services.

At 9 years of follow-up, statistically significant sex differences appear in all comorbidity categories except for SUD, ID and specific developmental disorders. Several categories of disorders usually appearing in late adolescence or adulthood (psychotic, bipolar/other affective, eating and personality disorders) were rarely registered during the first few years after ADHD diagnosis.

### Association between pharmacological treatment of ADHD and later comorbidity (LPM)

**Figure 2:**
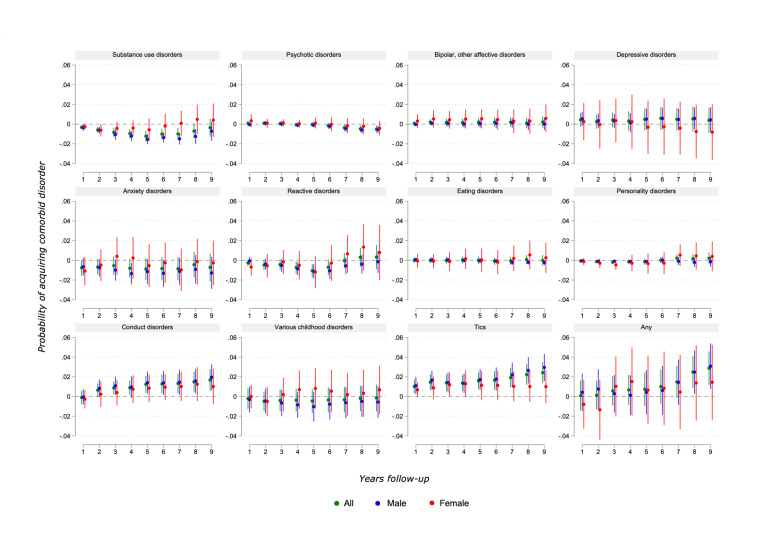
Results from linear probability models (LPM): associations between attention-deficit/hyperactivity disorder (ADHD) medication and psychiatric comorbidities. Patients diagnosed with ADHD in Norway in 2009–2011, aged 5–18 at time of diagnosis. Coefficient plots for regressions with 95% CIs. Adjusted for patient mix.

As shown in figure 2, LPM analyses returned no detectable associations, net of covariates, between ADHD medication and five categories of comorbidities: depressive, bipolar/other affective, eating, personality and various childhood disorders. (Adjusted and unadjusted coefficients with SEs can be found in online supplemental table S4.) Trends for males and females generally followed the same pattern, although estimates were less precise and trends less clear for the female subgroup. For females separately, no results were statistically significant at the 5% level.

For the male subgroup, and usually for the overall sample as well, several estimates were statistically significant at the 5% level. The results showed that medication usage was associated with a lower rate of comorbid SUD for 8 years after ADHD diagnosis, the association being strongest at year 5 (−1.2 percentage point (pp) reduction in the overall sample), and then gradually decreasing towards null, becoming statistically insignificant by year 9. CDs showed a steady increase over time with medication, gradually increasing from year 2 up to a maximum of 1.7 pp overall in year 9. Tic disorders showed a similar tendency, starting from year 1 and increasing by 2.4 pp overall in year 9. Psychotic disorders seemed to be reduced in years 7–9 with medication (up to −0.5 pp overall in years 8 and 9). Lastly, there was a tendency for anxiety and reactive disorders to temporarily decrease about 4–5 years after diagnosis (up to −0.7 pp for anxiety in males, and −0.7 to −1.1 pp for reactive disorders overall). Estimates for the any category were imprecise, as associations for different categories pulled in opposite directions, but in total a positive association was shown in years 8 and 9 (strongest in year 9 with 2.9 pp increase overall).

In summary, the results indicated that relative to the predicted values of the outcomes without pharmacological treatment, an increase from no medication to ‘full-time’ treatment with medication was associated with an incidence reduction in the overall sample of up to 51% for SUD (years 1 and 2), 36% for psychotic disorders (year 8), 22% for reactive disorders (year 5) and, in males, 22% for anxiety disorders (year 4); as well as an overall increase of up to 30% for CD (year 9), 48% for tics (year 9) and 7% for any disorder (year 9).

### Causal relations between pharmacological treatment of ADHD and later comorbidity (IV)

#### Assessment of the IV model

Prescription preferences varied considerably across CAMHS clinics. Variation was most pronounced over shorter durations of follow-up, but averaged out over longer durations of follow-up, so that differences in PP became too small to constitute an effective instrument. Hence, we estimated treatment effects for up to 4 years after ADHD diagnosis.

The first-stage F-statistic confirmed that the relevance assumption was met, as F values were 452, 219, 143 and 90 for follow-up durations of 1–4 years, respectively. This value should be >10 to minimise bias, or >104 for valid statistical inference.^25^ A balance test of the covariates for the IV produced joint F values of 5 or less for each year, supporting the independence assumptions. Figures illustrating these assumption tests for the current sample, as well as a more detailed methodological discussion of the IV, can be found in previous companion publications from our research group where we have applied the same IV method (ref ^26 27^, respectively).

#### Results of IV analyses

We performed IV analyses for eight categories (SUD, depressive, anxiety, reactive, CD, various childhood disorders, tics and any). The remaining categories were deemed unsuitable for IV analysis due to low numbers of outcome occurrences within our limitation of 4 years of follow-up (psychotic, in total 33 diagnoses; bipolar/other affective, 65; eating, 50; and personality disorders, 50).

**Figure 3:**
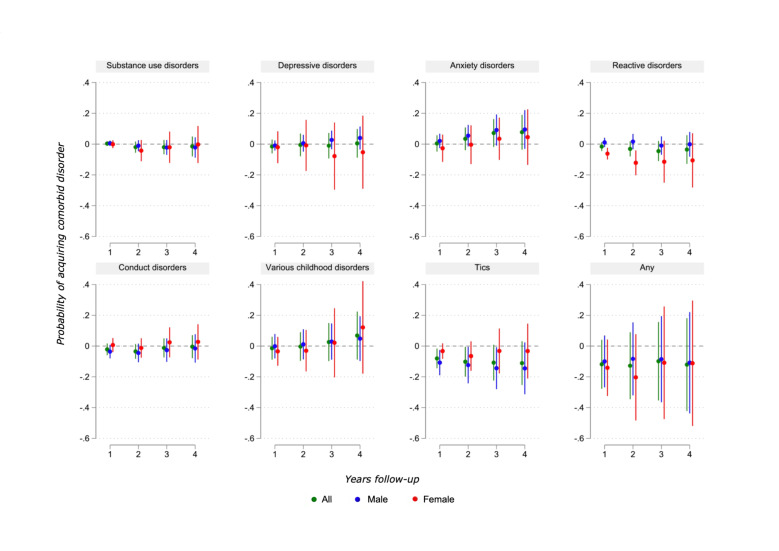
Results from instrumental variable (IV) analyses: effect estimates of attention-deficit/hyperactivity disorder (ADHD) medication on psychiatric comorbidities. Patients diagnosed with ADHD in Norway in 2009–2011, aged 5–18 at time of diagnosis. Coefficient plots for regressions with 95% CIs. Two-stage least squares (2SLS) estimates adjusted for patient mix.

Most IV analyses showed no statistically significant evidence for a causal effect of medication (figure 3; online supplemental table S5 gives coefficients and SEs). There was, however, support for a decrease in two outcomes for a limited number of years. For reactive disorders, the female subgroup showed a trend towards reduction, with statistically significant estimates at years 1–2 (−1.2 pp in year 2, which corresponds to eliminating the risk of the disorder entirely in this period). For tic disorders, there was support for the male subgroup in years 1–3 (−14.4 in year 3, indicating up to 89% decrease in rates). For anxiety, CD, tics, various childhood disorders and the ‘any’ category, the trends found by the IV and LPM analyses pointed in opposite directions.

### Robustness analyses

Results of analyses using Probit models corresponded to the results of our main analyses.

## Discussion

### Summary and interpretation of findings

We found psychiatric comorbidity to be common in individuals with ADHD, with more than half of patients being registered with at least one additional psychiatric diagnosis within 5 years of being diagnosed with ADHD. There were sex differences in most categories of comorbid diagnoses, for example, conduct and tic disorders were more prevalent in males, whereas affective, anxiety and stress-related disorders were more common in females. LPM showed significant associations between ADHD medication and incidence of several diagnostic categories, although strength of associations, direction and consistency during follow-up years varied. In contrast, causal analyses by IV, applicable for eight outcomes and 4 years of follow-up, mainly returned null findings. However, among females, medication nearly eliminated the incidence of reactive disorders over the first 2 years of follow-up. Likewise, for males, medication strongly decreased the incidence of tic disorders in the first 3 years.

Despite comprehensive data, ruling out unobserved confounding when investigating medication effects is challenging. For instance, the positive associations found between medication and CD and tics are unsurprising, as symptom severity is a predictor for both treatment and these outcomes. In the case of tics, positive associations may also be a result of stimulants directly triggering symptoms. A reduction in comorbidity with treatment, like we partially find for SUD and reactive disorders, on the other hand, could be caused by diverse mechanisms such as symptom alleviation of prescribed medications directly increasing coping and reducing the inclination to seek out self-medication; more indirectly by reducing exposure to negative life experiences; or simply be indicative of the patient generally receiving more follow-up in the healthcare system over time. Selection bias and reverse causality is a potential problem, as observed or assumed risk of comorbid disorders could influence a clinician’s treatment decisions (ie, patients more prone to SUD may be less likely to receive ADHD medication, and thus less likely to be included in the medicated group in the analyses).

The quasi-experimental IV method aims to correct for such unobserved confounding by exploiting plausibly as-if random variation in treatment; the trade-off being that we no longer estimate effects for the entire sample, but rather the smaller, latent subgroup of patients ‘on the margin of treatment’ who receive different treatment depending on the IV (here, due to PP). For this group, there was mostly no support for effects of medication on incidence of later comorbidity, and in several instances, trends were opposite compared with the LPM analyses. This could indicate confounding bias for the LPM results as mentioned above. However, results influenced by confounding factors may also be informative, as they reflect parts of the overall decision-making in a clinical setting. We thus think it is a strength of our study that we have applied both standard regression and causal inference methods.

An alternative explanation for discrepancies between the LPM and IV results may be that patients on the margin of treatment vary meaningfully from the overall group of treated patients. For example, girls with ADHD often present with less typical symptoms than boys, and may thus be more prone to variation in clinical practice and decision-making.^28^ The finding that medication for ADHD nearly eliminated the incidence of reactive disorders for females on the margin of treatment, up to 2 years, is clinically interesting and of potential importance in clinical decision-making. As reactive disorders are closely related to the individual’s response to life stressors, one explanation may be that medication for ADHD symptoms increases the individual’s general coping.

Moreover, IV addresses unobserved confounding and corrects for measurement error, which tends to bias LPM coefficients towards zero. Hence, IV may yield a more accurate larger effect. However, the IV estimates were considerably less precise than the LPM estimates and should consequently be interpreted with caution.

Both the standard regression (LPM) and causal inference (IV) method showed reduced effects of medication over time. For the LPM analyses, this may indicate different confounding factors for treatment initiation compared with treatment continuation or discontinuation. Studies have shown that about half of children and adolescents discontinue their medication after the first year of treatment, with or without later reinitiations, but we still know little about predictors and effects of such discontinuation.^29^ The observed reduction of variation in PP over time is interesting and may indicate that clinicians adapt their decisions over time with a ‘regression towards the mean’ effect.

### Strengths and limitations

The greatest strengths of this study are the comprehensive data, providing reliable descriptive statistics and an informative account of associations between pharmacological treatment and subsequent comorbidity in a full population sample followed up over a long time. Further, this study answers the call for studies covering a larger spectrum of diagnoses comorbid to ADHD, usage of ICD-10 criteria and females with ADHD,^14^ as well as exploration of whether ADHD treatment can decrease the risk of comorbid disorders.^4^ Being based on complete population data, the findings should be generalisable at least to children and adolescents living in comparable countries and with similar health systems.

A key strength to our design relative to other methods for causal inference (eg, within-subjects designs and propensity score matching) is that IV analysis can circumvent unobserved confounding. Moreover, IV analysis can correct for potential measurement error and reverse causality. While it is unclear whether the LATE estimates are representative for the general ADHD population, knowledge about medication effects for this subgroup is nonetheless valuable: by definition, the patients ‘on the margin’ represent the patients for whom clinicians most seem to vary in their evaluations of which treatment approach will serve the patient best, and where practice variations are greatest. While by definition we cannot know exactly who or how many the patients on the margin are, this group likely corresponds to those individuals that are increasingly being included in the patient pool as diagnosis and treatment rates rise.^30^ Knowledge about whether this expanded inclusion has benefit for the affected individuals is informative to the ongoing debates regarding varying diagnosis and treatment rates of ADHD.

We also note some limitations. Although comprehensive, our data for the LPM analyses lack some important potentially confounding variables, for example, measures of symptom load and psychosocial interventions. Further, registration practices may cause some variables to lack validity, as previously mentioned regarding sleep disorder diagnoses, or introduce lag, as we suspect in the case of SUD. If clinicians purposefully delay comorbid diagnosis, this represents a problem when aiming to investigate causal relations between treatment and diagnosis as an outcome. Other authors working with the same Norwegian register data have pointed out that comorbidity most likely is generally under-reported in these registries, and that patients may have diagnoses that are not listed in the proper register.^15^ At the same time, there may be a selection bias as those who eventually get more than one diagnosis are more likely to seek help as they experience a higher symptom load; also, receiving one diagnosis could in itself increase the chance of getting yet another.^4 15^ Furthermore, there may well be variation over time and/or between clinicians/clinics regarding differential diagnostic decisions or registration practices. Lastly, when conducting such a large number of tests, false-positive chance findings are likely to occur.

Although complete on a national population level, several outcome categories were so rarely registered during the first 4 years that they could not be included in the IV analyses, and LPM results for this period are based on low numbers of occurrences. Generally, our statistical precision was not sufficiently high for estimating informative effects with IV. IV is known for producing wide CIs and without sufficient power, this could cause a failure to recognise an actual effect.^31^ Because females made up less than a third of the ADHD sample, estimates were particularly imprecise for this subgroup. Furthermore, like all statistical methods, IV designs rest on a set of assumptions, not all of which can be empirically tested. Because *relevance* is the only assumption that can be verified, confidence in our results depends on appraising that the remaining assumptions have plausibly been met. Lastly, for outcomes usually occurring in late adolescence or adulthood, even a 9-year follow-up window is too short for this generally young patient sample—particularly for males, who are typically diagnosed with ADHD at an earlier age.

### Clinical implications

Clinicians should be aware that most children and adolescents with ADHD will be diagnosed with additional psychiatric disorders, and that pharmacological treatment may have protective effects on some of these comorbidities. However, although we did not find support for any harmful causal effect of ADHD medication on psychiatric comorbidity for patients on the margin of treatment, both benefits and risks should be monitored carefully in the follow-up of individual patients.

## Conclusion

This study, based on comprehensive population data with long-term follow-up and negligible attrition, shows that young individuals diagnosed with ADHD frequently present with at least one comorbid psychiatric disorder, in a pattern that varies by sex and with time. While analyses by conventional regression models indicate that pharmacological treatment is associated (sometimes positively, sometimes negatively) with the subsequent incidence of most categories of comorbid disorders, these associations are likely biased due to unobserved confounding. Causal inference analyses indicated a protective effect on reactive disorders among females and tic disorders in males for patients most prone to variation in prescription preference. These effects were significant in the first years of treatment, but no statistically significant evidence was found in the longer term. No causal effects of ADHD medication, neither protective nor harmful, were found for other comorbid disorder categories.

## Supplementary material

10.1136/bmjment-2024-301003 online supplemental file 1

## Data Availability

Data may be obtained from a third party and are not publicly available.
