# Supplementary material for: Effect of pharmacological treatment of attention-deficit/hyperactivity disorder on later psychiatric comorbidity: a population-based prospective long-term study
Source: BMJ Ment Health. 2024 Sep 19;27(1):e301003. doi: 10.1136/bmjment-2024-301003 (PMC11418547; doi:10.1136/bmjment-2024-301003)
Supplement: online supplemental file 1 [file bmjment-27-1-s001.docx]

**Table S1.** Diagnostic categories.

| **Block** | **Category** | **F-codes** |
| --- | --- | --- |
| F1 | Substance use disorders | F10.0-F19.9 |
| F2 | Psychosis | F20.0-F29.9 |
| F3 | Bipolar, other affective disorders | F30.0-F31.9; F34.0; F34.8-F34.9; F38.0; F38.8; F39.0 |
| F3, F9 | Depression, dystymia | F32.0-F33.9; F34.1; F38.1; F92.0** |
| F4, F9 | Anxiety disorders (phobias, GAD, OCD) | F40.0-F42.9; F92.8**; F93.0-F93.2; F94.0 |
| F4 | Reactive disorders (PTSD, adjustment, dissociation, somatization) | F43.0-F48.9 |
| F5 | Eating disorders | F50.0-F50.9; F98.2 |
| F6 | Personality disorders | F60.0-F61.0; F62.0-F62.9; F68.0-F69.0 |
| F7 | Mental retardation | F70.0-F79.9 |
| F8 | Pervasive developmental disorders | F84.0-F84.9 |
| F8 | Specific developmental disorders, other | F80.0-F83.0; F88.0, F89.0 |
| F9 | Conduct disorders | F91.0-F91.9; F92.0**, F92.8-F92.9**; F90.1* |
| F9 | Tic disorders | F95.0-F95.9 |
| F9 | Various childhood disorders | F92.9**; F93.3-F93.9; F94.1-F94.9; F98.0-F98.9; F99.0 |

**Note:** * counted as comorbidity if given following 90.0, 90.8, 90.9 as 90.1 in these cases represent an addition to the original diagnosis

(i.e., conduct disorder in addition to the initial ADHD diagnosis)

** combined diagnoses, counted in both relevant categories

**Table S2**. Complete list of covariates.

|  | **ADHD patients**  **(n = 8,051)** |
| --- | --- |
| ***Patient characteristics*** |  |
| Age at diagnosis, mean ± SD^1^ | 11.7 ± 3.4 |
| Male, n (%) | 5,566 (69.1) |
| Place of birth, n (%) |  |
| Norway | 6,263 (77.8) |
| Europe | 1,080 (13.4) |
| Outside Europe | 707 (8.8) |
| Comorbidity, no. (%) | 2,003 (24.9) |
| Child protective services intervention  prior to ADHD diagnosis, n (%) | 1,379 (17.13) |
|  |  |
| ***Family characteristics*** |  |
| Parents’ labor income (USD), mean ± SD^2^ |  |
| Mother: labor income | 28,374 ± 24,879 |
| Father: labor income | 54,900 ± 40,410 |
| Parents’ education level, n (%) |  |
| Mother: primary school | 2,640 (32.8) |
| high school | 3,437 (42.7) |
| university, short | 1,629 (20.2) |
| university, long | 221 (2.8) |
| Father: primary school | 2,561 (31.8) |
| high school | 3,849 (47.8) |
| university, short | 994 (12.4) |
| university, long | 316 (3.9) |
| Parents’ civil status, n (%) |  |
| Mother: married | 3,785 (47.0) |
| unmarried | 2,526 (31.4) |
| other | 1,604 (19.9) |
| Father: married | 3,767 (46.8) |
| unmarried | 2,356 (29.3) |
| other | 1,474 (18.3) |
|  |  |
| ***Catchment area characteristics*** |  |
| Population (0-65+ yrs.), mean ± SD | 32,913 ± 26,765 |
| Youth immigrants, % ± SD | 26.8 ± 10.5 |
| High school dropout, % ± SD | 25.6 ± 4.1 |
| Parents’ primary school education, % ± SD | 7.9 ± 4.6 |
| Parents married, % ± SD | 60.4 ± 6.3 |
| Parents’ labour income (USD), mean ± SD | 48,019 ± 7,192 |
|  |  |

**Note:** Individual level covariates, measured at baseline: sex, age, prevalent comorbid psychiatric diagnoses at time of ADHD diagnosis, country of birth, year of contact with CAMHS, child protection service intervention prior to ADHD diagnosis, and parents’ marital status.

Parents’ labour income and education level measured when the child was 6 years old.

Area level characteristics, collected for years 2009-2011: population size and high school dropout rates.

Information from the general population sample was used to develop aggregated measures for municipality-level rates of young immigrants, parents’ income and education level, and mothers’ marriage status.

**Table S3.** Cumulative incidence (%) of psychiatric comorbidity in ADHD patients, by sex.

|  | **Follow-up year** | | | |
| --- | --- | --- | --- | --- |
|  | *0* | *1* | *4* | *9* |
|  |  |  |  |  |
| **Any comorbidity** |  |  |  |  |
| Male | 38.09* | 43.53 | 51.35* | 60.73* |
| Female | 35.94* | 41.77 | 52.56* | 66.60* |
|  |  |  |  |  |
| **Substance use disorders** | |  |  |  |
| Male | 0.27 | 0.49 | 1.87 | 6.43 |
| Female | 0.08 | 0.36 | 2.21 | 7.00 |
|  |  |  |  |  |
| **Psychotic disorders** |  |  |  |  |
| Male | 0.02 | 0.09 | 0.41 | 1.10* |
| Female | 0.12 | 0.20 | 0.40 | 1.65* |
|  |  |  |  |  |
| **Bipolar, other affective disorders** | |  |  |  |
| Male | 0.11 | 0.20* | 0.43* | 1.11* |
| Female | 0.12 | 0.52* | 1.65* | 3.78* |
|  |  |  |  |  |
| **Depressive disorders** |  |  |  |  |
| Male | 1.15* | 1.67* | 3.47* | 8.14* |
| Female | 4.67* | 6.20* | 11.55* | 21.73* |
|  |  |  |  |  |
| **Anxiety disorders** |  |  |  |  |
| Male | 2.14* | 3.40* | 5.68* | 9.81* |
| Female | 3.86* | 5.23* | 9.90* | 20.04* |
|  |  |  |  |  |
| **Reactive disorders** |  |  |  |  |
| Male | 0.77* | 1.13* | 2.82* | 6.31* |
| Female | 1.65* | 2.54* | 6.12* | 15.49* |
|  |  |  |  |  |
| **Eating disorders** |  |  |  |  |
| Male | 0.07* | 0.14* | 0.25* | 0.43* |
| Female | 0.24* | 0.64* | 1.49* | 3.54* |
|  |  |  |  |  |
| **Personality disorders** | |  |  |  |
| Male | 0.00 | 0.02* | 0.23* | 1.20* |
| Female | 0.04 | 0.16* | 1.49* | 6.80* |
|  |  |  |  |  |
| **Intellectual disability** |  |  |  |  |
| Male | 1.35 | 1.51 | 1.94 | 2.73 |
| Female | 1.81 | 1.89 | 2.29 | 3.22 |
|  |  |  |  |  |
| **Pervasive developmental disorders** | |  |  |  |
| Male | 0.66 | 1.81* | 4.51* | 6.93* |
| Female | 0.32 | 1.01* | 2.49* | 4.67* |
|  |  |  |  |  |
| **Specific developmental disorders** | |  |  |  |
| Male | 16.94 | 19.26 | 22.44 | 25.60 |
| Female | 16.74 | 18.67 | 21.33 | 24.06 |
|  |  |  |  |  |
| **Tic disorders** |  |  |  |  |
| Male | 3.79* | 4.92* | 6.81* | 7.76* |
| Female | 1.65* | 1.97* | 2.66* | 2.94* |
|  |  |  |  |  |
| **Conduct disorders** |  |  |  |  |
| Male | 14.93* | 16.13* | 17.86* | 19.87* |
| Female | 9.09* | 9.86* | 11.67* | 14.45* |
|  |  |  |  |  |
| **Various childhood disorders** | |  |  |  |
| Male | 3.88 | 5.32 | 6.90 | 9.00* |
| Female | 3.66 | 4.59 | 7.16 | 10.99* |
|  |  |  |  |  |

**Note:** Year 0 signifies time of ADHD diagnosis.

* indicates a statistically significant sex difference at p<0.05

**Supplementary table S4**. Unadjusted and adjusted results of LPM analyses for the overall ADHD patient sample.

|  |  | | **Year 1** | | **Year 2** | | **Year 3** | | **Year 4** | | **Year 5** | | **Year 6** | | **Year 7** | | **Year 8** | | **Year 9** | |
| --- | --- | --- | --- | --- | --- | --- | --- | --- | --- | --- | --- | --- | --- | --- | --- | --- | --- | --- | --- | --- |
|  |  | | **Unadj.** | **Adjust.** | **Unadj.** | **Adjust.** | **Unadj.** | **Adjust.** | **Unadj.** | **Adjust.** | **Unadj.** | **Adjust.** | **Unadj.** | **Adjust.** | **Unadj.** | **Adjust.** | **Unadj.** | **Adjust.** | **Unadj.** | **Adjust.** |
| **Substance use** | | **Coeff.** | -.002 | -.003 | -.007 | -.006 | -.013 | -.008 | -.017 | -.010 | -.026 | -.012 | -.029 | -.010 | -.032 | -.010 | -.033 | -.007 | -.033 | -.004 |
|  | | **SE** | (.001) | (.001) | (.002) | (.002) | (.002) | (.002) | (.003) | (.002) | (.003) | (.003) | (.003) | (.003) | (.003) | (.003) | (.004) | (.004) | (.004) | (.005) |
|  | |  |  |  |  |  |  |  |  |  |  |  |  |  |  |  |  |  |  |  |
| **Psychotic** | | **Coeff.** | .001 | .001 | .001 | .001 | -.000 | .001 | -.002 | -.001 | -.002 | -.000 | -.004 | -.002 | -.007 | -.004 | -.008 | -.005 | -.010 | -.005 |
|  | | **SE** | (.001) | (.001) | (.001) | (.001) | (.001) | (.001) | (.001) | (.001) | (.001) | (.001) | (.001) | (.001) | (.002) | (.002) | (.002) | (.002) | (.002) | (.002) |
|  | |  |  |  |  |  |  |  |  |  |  |  |  |  |  |  |  |  |  |  |
| **Bipolar/** | | **Coeff.** | .001 | .001 | .002 | .002 | .001 | .002 | -.001 | .001 | -.001 | .002 | -.002 | .002 | -.003 | .002 | -.004 | .001 | -.004 | .002 |
| **other affective** | | **SE** | (.001) | (.001) | (.001) | (.001) | (.002) | (.002) | (.002) | (.002) | (.002) | (.002) | (.002) | (.002) | (.002) | (.002) | (.002) | (.003) | (.003) | (.003) |
|  | |  |  |  |  |  |  |  |  |  |  |  |  |  |  |  |  |  |  |  |
| **Depressive** | | **Coeff.** | .009 | .004 | .000 | .002 | -.006 | .004 | -.015 | .003 | -.020 | .005 | -.024 | .006 | -.030 | .005 | -.032 | .005 | -.034 | .004 |
|  | | **SE** | (.004) | (.004) | (.003) | (.004) | (.003) | (.004) | (.005) | (.005) | (.005) | (.006) | (.006) | (.006) | (.005) | (.006) | (.006) | (.006) | (.006) | (.006) |
|  | |  |  |  |  |  |  |  |  |  |  |  |  |  |  |  |  |  |  |  |
| **Anxiety** | | **Coeff.** | -.003 | -.008 | -.006 | -.007 | -.009 | -.005 | -.016 | -.008 | -.020 | -.009 | -.021 | -.008 | -.025 | -.009 | -.022 | -.004 | -.027 | -.007 |
|  | | **SE** | (.003) | (.004) | (.004) | (.004) | (.004) | (.005) | (.005) | (.005) | (.005) | (.006) | (.006) | (.006) | (.006) | (.006) | (.006) | (.007) | (.007) | (.007) |
|  | |  |  |  |  |  |  |  |  |  |  |  |  |  |  |  |  |  |  |  |
| **Reactive** | | **Coeff.** | -.000 | -.003 | -.004 | -.005 | -.009 | -.004 | -.016 | -.007 | -.021 | -.011 | -.021 | -.007 | -.020 | -.000 | -.019 | .003 | -.020 | .003 |
|  | | **SE** | (.002) | (.002) | (.003) | (.002) | (.003) | (.003) | (.003) | (.003) | (.003) | (.003) | (.004) | (.004) | (.004) | (.004) | (.005) | (.005) | (.006) | (.006) |
|  | |  |  |  |  |  |  |  |  |  |  |  |  |  |  |  |  |  |  |  |
| **Eating** | | **Coeff.** | .001 | .000 | -.000 | -.000 | -.000 | -.000 | -.000 | .000 | -.001 | -.000 | -.003 | -.001 | -.003 | -.001 | -.001 | .001 | -.002 | -.000 |
|  | | **SE** | (.001) | (.001) | (.001) | (.001) | (.002) | (.002) | (.002) | (.002) | (.002) | (.002) | (.002) | (.002) | (.002) | (.002) | (.002) | (.002) | (.002) | (.003) |
|  | |  |  |  |  |  |  |  |  |  |  |  |  |  |  |  |  |  |  |  |
| **Personality** | | **Coeff.** | -.000 | -.001 | -.002 | -.001 | -.003 | -.002 | -.004 | -.001 | -.006 | -.001 | -.006 | .000 | -.006 | .002 | -.009 | .002 | -.009 | .002 |
|  | | **SE** | (.000) | (.001) | (.001) | (.001) | (.001) | (.001) | (.001) | (.001) | (.002) | (.002) | (.002) | (.002) | (.002) | (.002) | (.002) | (.002) | (.003) | (.003) |
|  | |  |  |  |  |  |  |  |  |  |  |  |  |  |  |  |  |  |  |  |
| **Conduct** | | **Coeff.** | -.004 | -.001 | .004 | .006 | .008 | .009 | .009 | .009 | .012 | .012 | .012 | .013 | .013 | .013 | .014 | .015 | .016 | .017 |
|  | | **SE** | (.003) | (.003) | (.003) | (.003) | (.003) | (.003) | (.004) | (.004) | (.004) | (.004) | (.005) | (.005) | (.005) | (.005) | (.005) | (.005) | (.005) | (.005) |
|  | |  |  |  |  |  |  |  |  |  |  |  |  |  |  |  |  |  |  |  |
| **Tic** | | **Coeff.** | .007 | .010 | .014 | .015 | .016 | .014 | .020 | .014 | .025 | .016 | .028 | .016 | .031 | .019 | .035 | .022 | .037 | .024 |
|  | | **SE** | (.004) | (.003) | (.004) | (.004) | (.004) | (.004) | (.004) | (.004) | (.004) | (.004) | (.005) | (.005) | (.005) | (.005) | (.005) | (.005) | (.006) | (.005) |
|  | |  |  |  |  |  |  |  |  |  |  |  |  |  |  |  |  |  |  |  |
| **Various** | | **Coeff.** | -.004 | -.002 | -.005 | -.005 | -.005 | -.004 | -.004 | -.004 | -.005 | -.005 | -.004 | -.004 | -.004 | -.003 | -.002 | -.002 | -.001 | -.002 |
| **childhood dis.** | | **SE** | (.005) | (.005) | (.005) | (.005) | (.006) | (.006) | (.006) | (.006) | (.007) | (.006) | (.007) | (.006) | (.007) | (.007) | (.008) | (.006) | (.008) | (.007) |
|  | |  |  |  |  |  |  |  |  |  |  |  |  |  |  |  |  |  |  |  |
| **Any** | | **Coeff.** | .005 | .001 | -.001 | .001 | -.008 | .006 | -.013 | .007 | -.020 | .007 | -.022 | .010 | -.022 | .015 | -.015 | .025 | -.012 | .029 |
|  | | **SE** | (.007) | (.008) | (.007) | (.007) | (.008) | (.007) | (.008) | (.008) | (.009) | (.009) | (.008) | (.009) | (.008) | (.008) | (.009) | (.008) | (.009) | (.009) |

**Supplementary table S5**. IV results for the overall ADHD patient sample.

|  |  | **Year 1** | **Year 2** | **Year 3** | **Year 4** |
| --- | --- | --- | --- | --- | --- |
|  |  |  |  |  |  |
| **Substance use** | Coef | .003 | -.020 | -.020 | -.015 |
|  | SE | (.007) | (.019) | (.024) | (.033) |
|  |  |  |  |  |  |
| **Depressive** | Coef | -.015 | -.005 | -.010 | .005 |
|  | SE | (.023) | (.038) | (.042) | (.048) |
|  |  |  |  |  |  |
| **Anxiety** | Coef | .004 | .035 | .072 | .077 |
|  | SE | (.027) | (.038) | (.046) | (.058) |
|  |  |  |  |  |  |
| **Reactive** | Coef | -.016 | -.031 | -.045 | -.035 |
|  | SE | (.015) | (.025) | (.033) | (.048) |
|  |  |  |  |  |  |
| **Conduct** | Coef | -.022 | -.035 | -.012 | -.004 |
|  | SE | (.019) | (.025) | (.032) | (.038) |
|  |  |  |  |  |  |
| **Tic** | Coef | -.080 | -.102 | -.108 | -.111 |
|  | SE | (.033) | (.049) | (.059) | (.073) |
|  |  |  |  |  |  |
| **Various childhood dis.** | Coef | -.014 | -.003 | .026 | .068 |
|  | SE | (.038) | (.048) | (.063) | (.080) |
|  |  |  |  |  |  |
| **Any** | Coef | -.118 | -.128 | -.098 | -.121 |
|  | SE | (.081) | (.111) | (.130) | (.154) |

Adjusted for baseline covariates.
